# *Lutzomyia longipalpis* Saliva Induces Heme Oxygenase-1 Expression at Bite Sites

**DOI:** 10.3389/fimmu.2018.02779

**Published:** 2018-11-28

**Authors:** Nivea F. Luz, Thiago DeSouza-Vieira, Waldione De Castro, Aislan Carvalho Vivarini, Lais Pereira, Riam Rocha França, Paulo S. Silveira-Mattos, Diego L. Costa, Clarissa Teixeira, Claudio Meneses, Viviane S. Boaventura, Camila I. de Oliveira, Ulisses Gazos Lopes, Naomi Aronson, Bruno B. Andrade, Claudia I. Brodskyn, Jesus G. Valenzuela, Shaden Kamhawi, Valeria M. Borges

**Affiliations:** ^1^Gonçalo Moniz Institute, Oswaldo Cruz Foundation, Salvador, Brazil; ^2^Vector Molecular Biology Section, Laboratory of Malaria and Vector Research, National Institute of Allergy and Infectious Diseases, National Institutes of Health, Rockville, MD, United States; ^3^Laboratory of Molecular Parasitology, Center of Health Science, Carlos Chagas Filho Biophysics Institute, Federal University of Rio de Janeiro, Rio de Janeiro, Brazil; ^4^Faculdade de Medicina da Bahia, Universidade Federal da Bahia, Salvador, Brazil; ^5^Immunobiology Section, Laboratory of Parasitic Diseases, National Institute of Allergy and Infectious Diseases, National Institutes of Health, Bethesda, MD, United States; ^6^Oswaldo Cruz Foundation (Fiocruz-PI), Teresina, Brazil; ^7^Infectious Diseases Division, Uniformed Services University of the Health Sciences, Bethesda, MD, United States; ^8^Multinational Organization Network Sponsoring Translational and Epidemiological Research Initiative, Fundação José Silveira, Salvador, Brazil

**Keywords:** *Lutzomyia longipalpis*, sand fly bite, saliva, skin, macrophages, heme oxygenase-1, Nrf2

## Abstract

Sand flies bite mammalian hosts to obtain a blood meal, driving changes in the host inflammatory response that support the establishment of *Leishmania* infection. This effect is partially attributed to components of sand fly saliva, which are able to recruit and activate leukocytes. Our group has shown that heme oxygenase-1 (HO-1) favors *Leishmania* survival in infected cells by reducing inflammatory responses. Here, we show that exposure to sand fly bites is associated with induction of HO-1 *in vivo*. Histopathological analyses of skin specimens from human volunteers experimentally exposed to sand fly bites revealed that HO-1 and Nrf2 are produced at bite sites in the skin. These results were recapitulated in mice ears injected with a salivary gland sonicate (SGS) or exposed to sand fly bites, indicating that vector saliva may be a key factor in triggering HO-1 expression. Resident skin macrophages were the main source HO-1 at 24–48 h after bites. Additionally, assays *in vivo* after bites and *in vitro* after stimulation with saliva both demonstrated that HO-1 production by macrophages was Nrf2-dependent. Collectively, our data demonstrates that vector saliva induces early HO-1 production at the bite sites, representing a major event associated with establishment of naturally-transmitted *Leishmania* infections.

## Introduction

The leishmaniases are a group of diseases caused by protozoan parasites from more than 20 *Leishmania* species (1). There are three main forms of the disease: visceral leishmaniasis (VL, also known as kala azar), cutaneous leishmaniasis (CL), and mucosal leishmaniasis (MCL). While CL is the most common form of the disease, VL is the most serious and can be fatal if untreated. Most VL cases occur in Brazil, East Africa and in South-East Asia with an estimated 50,000–90,000 new cases occuring worldwide each year (2).

Phlebotomine sand flies (Diptera: Phlebotominae) are blood feeding insects of medical importance that transmit parasites of the genus *Leishmania*. Natural transmission of *Leishmania* to humans occurs during blood acquisition by female phlebotomine sand flies. As the sand fly bites, it introduces saliva into the vertebrate host dermis alongside metacyclic infective promastigotes and other vector-derived factors (3–6). Studies have demonstrated that saliva promotes *Leishmania* infections. *L. major* coinjected with *Lutzomyia longipalpis* or *Phlebotomus papatasi* saliva resulted in a more severe disease reflected by larger lesions when compared with a group of mice receiving parasites alone (7). This initial observation was supported by additional studies demonstrating the enhanced infectivity of *L. major* when coinoculated with saliva from the sand fly *L. longipalpis* (8, 9). Apart from antihemostatic properties, sand fly saliva is chemotactic for different immune cells, such as macrophages (10), neutrophils (11), dendritic cells (12), and lymphocytes (13). In addition, many cell types, including monocytes, interact with sand fly saliva, thereby modifying inflammatory processes at the blood feeding site (3). It has been proposed that such effects on the host immune system contribute to increased parasite loads in mice exposed to sand fly bites compared to animals infected through needle injection (3). The specific mechanism underlying the effect of vector saliva on the host immune response is not fully understood.

Heme oxygenase-1 (HO-1) is a key enzyme triggered by cellular stress, exhibiting cytoprotective, antioxidant, and anti-inflammatory properties (14). HO-1 is the rate-limiting enzyme in the catabolism of heme. It breaks down the porphyrin ring to yield equimolar amounts of biliverdin, free iron (Fe^+2^), and carbon monoxide (CO) (15). Pharmacological induction of HO-1 or administration of the end products of its activity can exert therapeutic effects in a variety of immune-mediated inflammatory diseases (16). The transcriptional induction of HO-1 occurs in response to multiple forms of cellular stress and tissue damage. Oxidative stress activates the transcription factor nuclear factor erythroid 2-related factor-2 (Nrf2), which in turn binds antioxidant response element (ARE) enhancers and induces expression of protective antioxidant genes including HO-1. Activation of Nrf2 requires its translocation to the nucleus and binding to ARE enhancer motifs (17, 18). In addition to Nrf2, IL-10 has been shown to directly mediate HO-1 expression (19). Importantly, IL-10 is highly upregulated in the skin up to 18 h after sand fly bites (6).

Previous studies from our group have shown that HO-1 favors *Leishmania* infection (20, 21). Based on our findings, we have proposed that HO-1 plays two major roles following *Leishmania* infection: (1) it prevents host cell damage, and (2) it decreases the ability of the host to limit intracellular growth of the parasite. Of note, we have also found that patients with VL presented higher systemic concentrations of HO-1 than healthy individuals, and that the levels of this enzyme substantially decreased after leishmanicidal treatment, suggesting that HO-1 is a potential biomarker of active VL (21, 22).

In the present study, we first demonstrated that experimental exposure to sand fly bites in humans triggers robust HO-1 protein expression *in situ*, in the skin, but not in blood. Further experiments in mice revealed that experimental exposure of mice ears to sand fly bites induced Nrf2-dependent HO-1 production by resident macrophages after 24–48 h. Similarly, injection of saliva induced HO-1 production in the ear dermis. Lastly, *in vitro* assays using macrophage cell lines showed that saliva drives Nrf2 translocation to the nucleus and ARE activation, leading to augmented HO-1 expression. Together, our findings identified HO-1 as a novel inflammatory mediator triggered by saliva deposited into skin during sand fly bites. This further facilitates our understanding of the early events during *Leishmania* infection that contribute to the successful establishment of the pathogen and onset of leishmaniasis.

## Results

### Experimental exposure to *lutzomyia longipalpis* bites induces *in situ* expression of HO-1 and Nrf2 in humans

**Figure 1:**
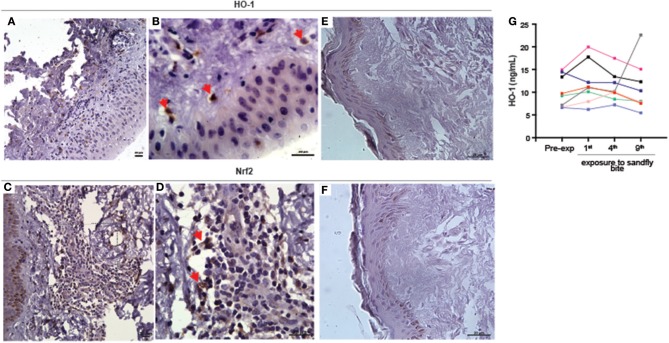
Experimental exposure to *Lutzomyia longiplapis* sand flies induces HO-1 and Nrf2 protein expression at bite sites in healthy human volunteers. **(A,B,D,E)** Immunohistochemistry of paraffin-embedded sections of skin specimens obtained by biopsy of the bite sites 48 h after sand fly exposure. **(A)** HO-1 staining in sand fly-exposed skin, 400X. **(B)** Higher magnification from image **(A)** (1000X). **(C)** Nrf-2 staining in sand fly-exposed skin, 400X. **(D)** Higher magnification from image **(C)** (1000X). **(E)** HO-1 staining in unbitten skin (1000X). **(F)** Nrf-2 staining in unbitten skin (1000X). Bar, 20 μm. Red arrows point to cells positively stained with HO-1 or Nrf2. Digital images 400 × (lower magnification) or 1000X (higher magnification) were captured using a Nikon E600 microscope and an Olympus Q-Color 1 digital camera with the Image Pro Plus software. **(G)** Plasma HO-1 levels in individuals (*n* = 7) before and after nine exposures to sand fly bites were measured by ELISA. Plasma samples were collected 48 h after each exposure to sand fly bites. Each line represents one individual volunteer. Data represent individual values and were compared using the Kruskal-Wallis test (*p* = 0.08).

Having previously demonstrated that HO-1 is induced during *Leishmania* infection and identified its association with active VL (21), we asked whether it plays a part in the early stages of parasite establishment after vector-transmission. Immunohistochemistry of paraffin-embedded skin sections, obtained 48 h after the last of several experimental exposures of human volunteers to sand fly bites, revealed that both HO-1 (Figures 1A,B and Supplemmentary Figure 1A) and Nrf2 (Figures 1C,D, Supplemmentary Figure 1B) were produced in the dermis *in situ*. Expression of both HO-1 and Nrf2 was not detected in control specimens of skin not bitten by sand flies taken from the same individuals (Figures 1E,F).

Next, we tested whether circulating concentrations of HO-1 protein levels in plasma of the human volunteers is also altered after exposure to sand fly bites. We observed that levels of HO-1 were undistinguishable between samples collected prior to exposure to bites and after up to nine repeated exposures (Figure 1G). Thus, HO-1 induction by bites of *L. longipalpis* in humans is restricted to the skin and is not followed by changes in its systemic levels. The real but focal HO-1 response driven by the sand fly bites is reinforced by the fact that the biopsies came from volunteers whose serum values remained unchanged (shown in blue and purple lines in Figure 1G).

### Bites of *lutzomyia longipalpis* induce Nfr2-dependent HO-1 production in resident skin macrophagess

**Figure 2:**
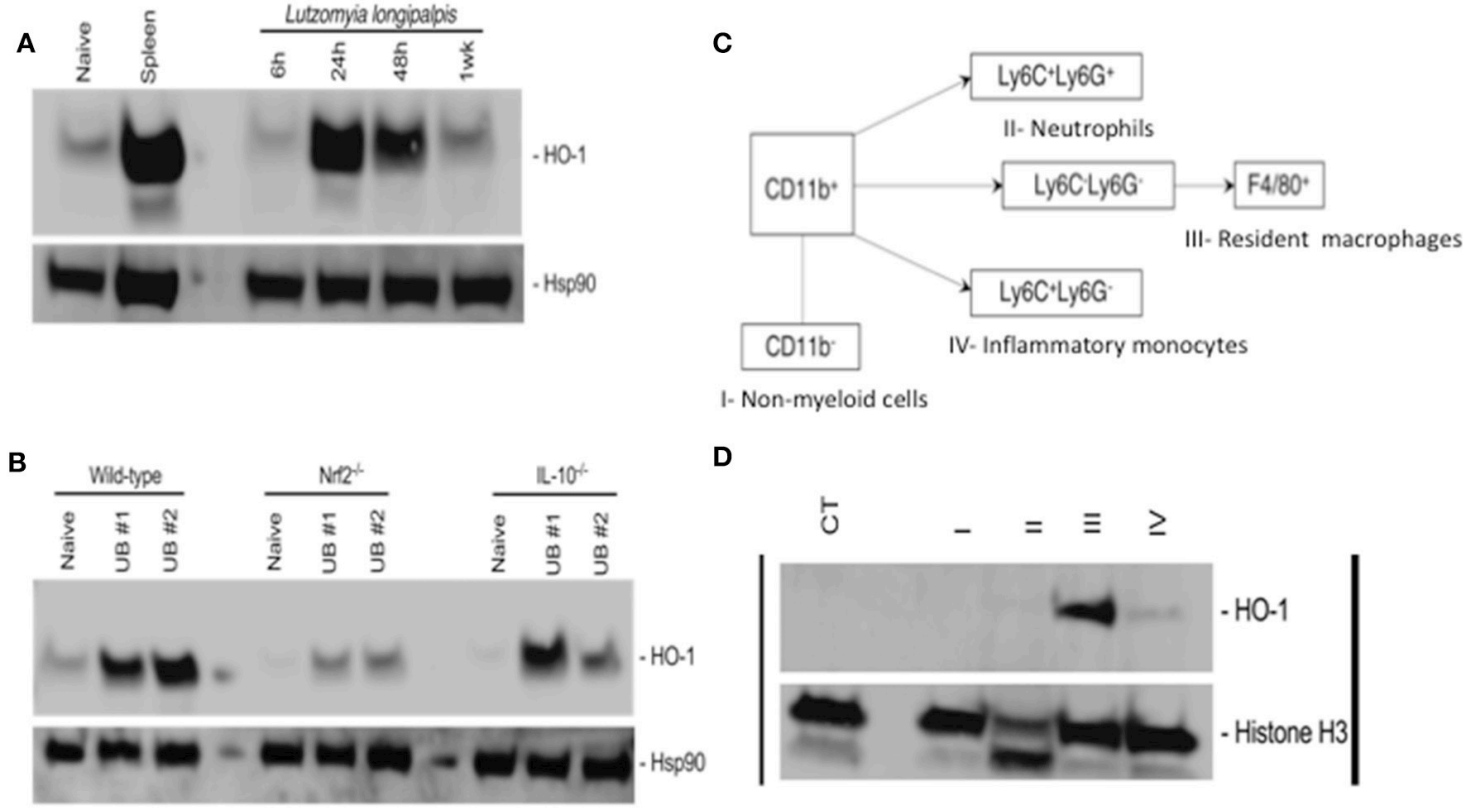
Bites of *Lutzomyia longipalpis* sand flies induce Nfr2-dependent HO-1 protein expression by resident macrophages in mice. Ears of C57BL/6 mice were exposed to bites of 20 uninfected *L. longipalpis* sand flies (UB, uinfected bite) and tissue was processed for total protein extraction before or after sorting by flow cytometry. **(A,B)** Western blots of total protein extracts from mice ears. Hsp90 was used as a loading control. **(A)** kinetics of HO-1 expression following sand fly bites. Total protein extract from a mouse spleen was used as a positive control. **(B)** HO-1 expression in wild-type, Nrf2 knockout (Nrf2^−/−^) and IL-10 knockout (IL-10^−/−^) mice 24 h after exposure to sand fly bites. **(C,D)** Ear cells recovered 24 h after sand fly bites were stained with antibodies for population sorting by flow cytometry prior to total protein extraction. **(C)** Gating strategy for sorting populations of non-myeloid cells (I), neutrophils (II), resident macrophages (III) and inflammatory monocytes (IV). **(D)** Western blot of total protein extract from populations sorted according to **(C)**. Naïve total ear cells were used as a negative control (CT). Histone H3 was used as a loading control. Blots are representative of two to three independent experiments.

To investigate whether sand flies also induce HO-1 during blood feeding on mice, we exposed 20 *Lu. longipalpis* adult females to mice ears and followed the kinetics of HO-1 production in host skin by western blot. Compared to naïve, unbitten, skin, HO-1 protein levels peaked at 24 h after sand fly bites and remained high up to 48 h before returning to basal levels at 1 week (Figure 2A). Of note, levels of HO-1 were comparable to controls at 6 h after sand fly bites. No differences were observed in the sand fly feeding score among the groups (Supplemmentary Figure 2A). Next, we assessed the involvement of Nrf2 or IL-10 in HO-1 induction at 24 h after sand fly bites using animals genetically deficient in either of these molecules. In contrast to wild-type and IL-10^−/−^ animals, HO-1 induction was substantially diminished exclusively in Nrf2^−/−^ animals (Figure 2B). No differences were observed in the sand fly feeding score among the groups (Supplemmentary Figure 2B).

**Figure 3:**
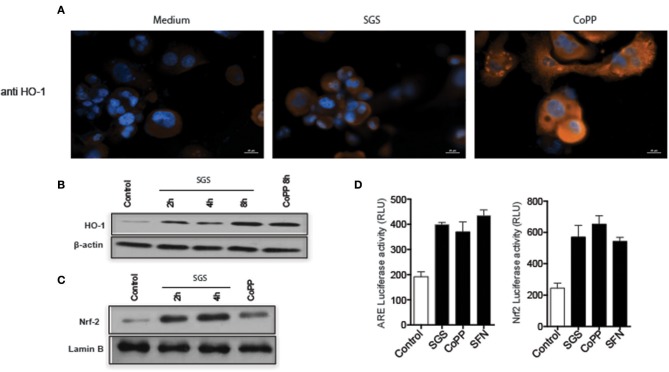
*Lutzomyia longipalpis* saliva activates ARE, nuclear translocation of Nrf2, and HO-1 expression in human macrophages. **(A)** THP-1 derived macrophages were plated on coverslips and stimulated with either the equivalent of one pair of salivary gland sonicate (SGS) per well or 30 μM CoPP (cobalt protoporphyrin IX) for 24 h, then stained for HO-1. Cells were counter-stained with 1 μg/mL of DAPI to visualize the nucleus. Orange and Blue colors indicate positive staining for HO-1 and nuclei, respectively. Bar, 20 μm. Digital images (400 × original magnification) were captured on a Nikon E600 microscope coupled to an Olympus Q-Color 1 digital camera, and visualized using Image Pro Plus software. **(B,C)** THP-1 macrophages were treated with one pair SGS per well, followed by extraction of total protein, and detection by Western blot. **(B)** HO-1. **(C)** Nrf-2. β-actin and Lamin B were used as loading controls. **(D)** RAW 264.7 cells were transiently transfected with p3xARE- or pNrf2-promoterluciferase reporter plasmids constructs and treated with 30 μm CoPP or 10 mM DL-sulforaphane (SFN) or one pair SGS per well for 24 h post-transfection. Whole-cell lysates were analyzed for luciferase activity of p3xARE and Nrf2. Data are representative of three independent experiments.

To identify the source of HO-1, we exposed mice ears to bites of 20 sand flies and recovered the cells 24 h later. Ear cells were sorted into non-myeloid cells, neutrophils, resident macrophages and inflammatory monocytes according to the gating strategy shown in Figure 2C and Supplemmentary Figure 3. Western blots of the total protein extracted from each population demonstrated that HO-1 is mainly produced by resident macrophages with a minor contribution from inflammatory monocytes (Figure 2D). Taken together, our data shows that sand fly bites trigger Nrf2-dependent expression of HO-1 by resident macrophages in host skin.

### SGS induces HO-1 expression in mice and in human macrophages

**Figure 4:**
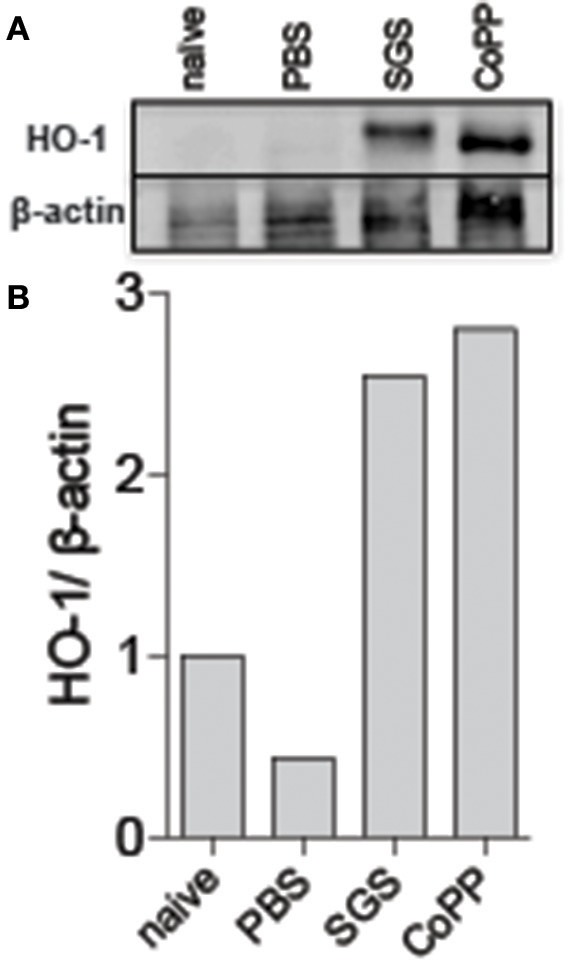
*Lutzomyia longipalpis* saliva induces HO-1 protein expression at bite sites in mice. C57BL/6 mice were injected intradermally in the ear with the equivalent of 1 pair of *L. longipalpis* salivary gland sonicate (SGS). **(A)** Western blot of whole-tissue extract 24 h after sand fly exposure. β-actin was used as a loading control. **(B)** Optical density of the bands in **(A)** was quantified using ImageJ software. Data are presented as the ratio of HO-1 to β-actin. Data are representative of two independent experiments.

We next tested whether sand fly saliva, deposited into host skin during blood feeding, could be the component contributing to the increased production of HO-1 in the dermis. Mice ears challenged intradermally with SGS exhibited high levels of HO-1 compared to animals challenged only with PBS at 24 h post-injection (Figures 3A,B). To verify if saliva was acting directly on macrophages, we conducted immunocytofluorescence assays using human THP-1 derived macrophages. Macrophages incubated with SGS induced a positive intracellular staining for HO-1 (Figure 4A). This finding was further confirmed using a WB assay, which also revealed that HO-1 induction *in vitro* occurred as early as 2 h post incubation of cells with SGS and was sustained up to 8 h later (Figure 4B). Together, these results demonstrate that HO-1 is robustly induced by SGS *in vivo* and *in vitro*.

Expression of HO-1 is described to be dependent on induction of the canonical antioxidant response element (ARE), which is recognized by the transcription factor Nrf2, which translocates from the cytosol to nucleus when activated (23). To confirm that the ARE/Nrf2 pathway is involved in SGS-driven HO-1 induction in our system, we evaluated Nrf2 protein expression in nuclear extracts from THP-1 macrophages incubated with SGS. As expected, Nrf2 expression was substantially increased as early as 2 h post incubation (Figure 4C). Participation of this pathway was further illustrated by using a luciferase reporter assay. In this experimental system, plasmid constructs containing the canonical ARE promoter response element or the Nrf2 promoter are transfected into RAW264.7 cells. Using this approach, we found that ARE and Nrf2 luciferase activities were significantly increased after incubation with SGS (Figure 4D). These findings reveal that SGS induces HO-1 in cells through activation of the ARE-Nrf2 pathway.

## Discussion

Modification of host inflammatory responses at the site of the sand-fly bite is critical for successful blood feeding (24), but it also plays an important role in the establishment of *Leishmania* infection (25). It is also well-known that experimental exposure to *Leishmania*-infected sand fly bites causes more severe disease associated with higher parasite loads in mice, whereas needle injection of *Leishmania* alone results in milder outcomes (3). Although several mechanisms have been proposed to explain this discrepancy (26), the exact pathway directly associated with the enhancing effect of *Leishmania* transmission by sand fly bites on infection has not been fully characterized. The results from our study adds to our current knowledge of this topic by demonstrating that sand fly saliva robustly induces HO-1 in the host dermis at the bite site during blood feeding.

In the present study, we observed a significant induction of HO-1 expression in the dermis of human volunteers experimentally exposed to *L. longipalpis* bites. HO-1 induction was localized to the bite site with no change in HO-1 serum levels after several repeated exposures of individuals to sand fly bites. Thus, the sand fly bite alone does not trigger systemic inflammation, not surprising given the relatively small area of tissue damage at the bite site. Of note, only two volunteers consented to provide skin biopsies for analysis. Nevertheless, both biopsies showed a clear induction of both HO-1 and Nrf2. Further, induction of HO-1 in human macrophages *in vitro* as well as its induction in mice reinforce the results obtained from human biopsies.

HO-1 is thought to be an intracellular enzyme (27), and its detection in plasma would indicate substantial tissue damage. In this scenario, the release of HO-1 occurs after cellular lysis during robust inflammation associated with infectious diseases such as VL (21, 28, 29), tuberculosis (30) and malaria (31, 32). For sand fly bite-induced HO-1 expression in the skin, our data demonstrates that it is triggered in resident skin macrophages by saliva and as such could play a critical role in the early establishment of *Leishmania* infection. Production of HO-1 in the dermis microenvironment may counterbalance the host anti-parasite effector mechanisms such as free radial production. Additional studies are warranted to directly test this hypothesis.

The direct induction of HO-1 by sand-fly bite is intriguing. During blood feeding the vector probes in search for capillaries to produce the hemorrhagic pool upon which it feeds. Therefore, it is possible that mechanical or inflammation-driven (25) tissue damage could result in release of heme from damaged cells. Heme is an extremely toxic molecule, which can induce significant oxidative stress and cell death (33). Indeed, we have previously published that heme induces inflammatory cell death in neutrophils (34) and in macrophages (22) in the context of *Leishmania* infection *in vitro*. Importantly, heme is the canonical substrate of HO-1 (35), and accumulation of heme in tissues is directly associated with induction of this enzyme (36). Thus, it is reasonable to hypothesize that an increase in heme availability in the dermis at the site of a sand-fly bite could be the trigger for the local induction of HO-1 to minimize the deleterious effects of free heme during blood feeding. However, results from our *in vitro* and *ex vivo* experiments clearly demonstrate that HO-1 is directly induced by sand-fly saliva. This is an important observation because it indicates that HO-1 production may not be due to non-specific inflammation from tissue injury caused by the bite. Alternately, both tissue damage and saliva could be contributing to the robust induction of HO-1 after bites. Further studies are needed to elucidate this aspect of the innate immune response to sand fly bites. Either way, sand fly bite-mediated induction of HO-1 in the dermis early after sand fly bites is likely to support the establishment of *Leishmania* in the host. The specific molecule from saliva responsible for HO-1 induction remains to be determined.

One pathway for the induction of HO-1 requires activation and nuclear translocation of Nrf2 (37). This transcription factor is activated during cellular stress and it binds to the ARE motif in the nucleus, which promotes transcription of HO-1 mRNA (38). Other triggers of HO-1 expression have also been reported including IL-10 that induces HO-1 expression through activation of the signal transducer and activator of transcription 3 (STAT-3) (19). IL-10 is induced by saliva (26) and is present at high levels in mice ears after bites of *L. longipalpis* sand flies (6). Nevertheless, our data confirms that saliva directly induces HO-1 in cells through activation of the ARE-Nrf2 pathway and not indirectly through its induction of IL-10. Previous studies from our group have described that *Leishmania* infection interferes with macrophage signaling pathways triggering Nrf2 activation (23). As such, the induction of Nrf-2 by both sand fly saliva and *Leishmania* could serve as a mechanism to deal with the altered oxidative state of infected macrophages resulting in increased susceptibility to *Leishmania* infection.

Altogether, our findings demonstrate that sand fly saliva induces HO-1 production at the bite site. This suggests that in addition to its importance in disease pathogenesis, HO-1 may play a critical role in the early establishment of *Leishmania* parasites after vector-transmission.

## Materials and methods

### Study subjects

Healthy human volunteers were experimentally exposed to bites of 10 laboratory reared *Lutzomyia longipalpis* sand flies as approved by the Institutional Review Boards of the Walter Reed Army Medical Center, the Uniformed Services University of the Health Sciences, and the National Institute of Allergy and Infectious Diseases (NIAID). All clinical investigations were conducted in accordance with the Declaration of Helsinki revision 2013 principles. Written informed consent was obtained from all participants.

### Mice

C57BL/6, Nrf2^−/−^ and IL-10^−/−^ mice were obtained from Jackson Laboratories (Bar Harbor, ME). Animals were housed under pathogen-free conditions at the NIAID Twinbrook animal facility in Rockville, Maryland. Animal experimental procedures were reviewed and approved by the Care and Use Committee of the National Institute of Allergy and Infectious Diseases (NIAID) and the Gonçalo Moniz Institute (IGM), FIOCRUZ, Salvador, Bahia-Brazil. The Animal Care and Use program at NIAID DIR and IGM-FIOCRUZ comply with the Guide for the Care and Use of Laboratory Animals and with the NIH OACU and ARAC guidelines.

### Cell lines and culture

Human monocytic leukemia cell line THP-1 (ATCC:TIB202TM) and mouse macrophage leukemia cell line RAW 264.7 (TIB-71; American Type Culture Collection (ATCC), Manassas, VA, USA) were maintained in RPMI or DMEM medium with high glucose, respectively (Vitrocell Embriolife, Campinas, SP, Brazil) supplemented with 10% heat-inactivated fetal bovine serum (Sigma-Aldrich, St. Louis, MO, USA). THP-1 cells were differentiated to macrophages with 40 ng/mL of PMA for 3 days. Afterwards, the cells were washed 3 times with PBS and incubated with fresh medium for an additional 3 days. To induce the activation of Nrf2, 10 mM SFN (sulforaphane) were used as positive controls.

### Sand fly rearing and salivary gland homogenate preparation

*L. longipalpis* sand flies were collected in Mali (39, 40) and reared at the Laboratory of Malaria and Vector Research (LMVR), NIAID. Salivary glands were dissected from 50, 5–7-day old colonized female *L. longipalpis* sand flies. The salivary glands were pooled, aliquoted, and used as needed to perform *in vitro* experiments. Salivary gland sonicate (SGS) was prepared by ultrasonication, followed by centrifugation at 10,000 g for 3 min at 4°C. Supernatants were collected and dried using a vacuum concentrator (Thermo, Asheville, NC). SGS were shipped to the Gonçalo Moniz Institute (IGM-FIOCRUZ, Salvador, Bahia-Brazil) and rehydrated with ultra-pure water (KD-Medical, Columbia, MD) immediately before use.

### Exposure of human volunteers to uninfected sand flies

A secure, custom-designed Plexiglas capsule (Precision Plastics, Beltsville, MD) with a meshed surface was used in the exposure experiments. Healthy *Lutzomyia* saliva antibody negative volunteers, 18–50 years old, were exposed to bites of 10 colony-reared female sand flies on a bi-weekly basis for the first 2 months (± 1 week) and once every 2 months (± 1 week) for the following 18 months. The capsule was strapped to the upper arm of each individual for 20 min and covered with a dark fabric to simulate nighttime conditions. All sand flies were accounted for and killed after each exposure. Two of 15 subjects volunteered 2 mm dermal specimens obtained by biopsy 48 h post-bite after the final exposure to sand flies.

### Exposure of mice to uninfected sand flies

Mice were anesthetized i.p. with a combination of 150 mg/kg ketamine and 20 mg/kg xylazine. Both mouse ears were placed inside vials containing 20 *L. longipalpis* females. Sand flies were allowed to feed for 1 h in the dark. Feeding counts were obtained by counting fully blood fed flies using a stereoscope.

### Immunodetection of HO-1 and Nrf2 in skin specimens obtained by biopsy and THP-1 cells

A 2-mm punch biopsy was obtained from the bite site of 2 volunteers 48 h after the 9th exposure to *L. longipalpis* sand flies. Normal skin samples was obtained from healthy individuals by plastic surgery. Biopsied samples were stored in 10% buffered formalin solution, and paraffin-embedded and sectioned (Histoserv, MD) for histological staining.

For immunohistochemistry, primary antibodies (diluted at 1:100) against HO-1 (Enzo, ADI-SPA-896-J, Ann Harbor, MI) and Nrf2 (Abcam, ab31163, Cambridge, MA) were used. Biotinylated anti-mouse, for HO-1 staining, and anti-rabbit, for Nrf2 staining (Dako) at 1:500 dilution were used as a secondary antibodies, followed by the addition of streptavidin-horseradish peroxidase. Slides were then counterstained with hematoxylin and eosin. Isotype control staining for HO-1 and Nrf-2 antibodies are shown in Supplemmentary Figure 4.

For immunofluorescence analysis, THP-1 cells (5 × 10^5^) were added to wells containing phorbol myristate acetate (PMA) for differentiation, plated on coverslips and allowed to adhere for 3 days and left for an additional 3 days. Cells were stimulated with SGS (equivalent to 1 pair/well) or CoPP (30 μM); Cobalt protoporphyrin IX (CoPP) (Frontier Scientific, Logan, UT) is a pharmacologic inducer of HO-1. CoPP was dissolved in 0.1N NaOH and RPMI 1640 medium and adjusted to concentrations of 30 μM for *in vitro* assays. After 24 h, cells were fixed in 4% paraformaldehyde (Sigma), followed by permeabilization with 0.1% Triton X-10, and blocking with 1% BSA. Primary antibody against HO-1 overnight. Cells were then incubated with the secondary antibody, Texas Red® anti-mouse IgG (H+L) (1:3,000) (Vector Laboratories, Burlingame, CA), followed by nuclear staining with 1 μg/mL of DAPI. A control staining protocol employing only the secondary antibody (i.e., no primary antibody) was implemented following the same procedure described above. Coverslips were analyzed by the fluorescence microscopy Nikon E600, and digital images were obtained using an Olympus Q-Color 1 digital camera with the Image Pro Plus software.

### HO-1 quantification by ELISA

Seven out of 15 volunteers completed nine exposures to sand fly bites. Serum samples were obtained from each individual before (a) and at 1, 4, and 9 weeks after exposure to *L. longipalpis* sand flies. Serum levels of HO-1 were measured using a commercially available ELISA kit (Enzo Life Sciences, Farmingdale, NY, USA).

### Immunoblotting

THP-1 cells (1 × 10^6^ cells) were washed twice with ice-cold PBS and then lysed in 100 μl of lysis buffer (50 mM Tris-HCl, pH 7.5, 5 mM EDTA, 10 mM EGTA, 50 mM NaF, 20 mM β-glycerophosphate, 250 mM NaCl, 0.1% Triton X-100, 1 μg/ml BSA and a 1:100 dilution of protease inhibitor cocktail, Sigma-Aldrich, St. Louis, MO, USA) for total protein extraction. For nuclear protein extraction, after infection and/or treatment, the cells were washed twice with 1x PBS and then lysed with 100 μL of buffer A (HEPES 10 mM pH 7.9. 10 mM KCl, 0.1 mM EDTA, 0.1 mM EGTA, NP-40 0,25% (v/v); cocktail of protease inhibitors) for 10 min on ice. The lysed cells were centrifuged at 14,000 g for 1 min at 4°C, and the pellet was resuspended in 60 μL of buffer C (20 mM HEPES pH 7.9, 0.4 M NaCl, 1 mM EDTA, 1 mM EGTA, 20% glycerol, protease inhibitor cocktail) and incubated on ice for 20 min. The lysate was centrifuged at 14,000 g for 5 min, and the supernatant containing nuclear proteins was collected in a new tube. The protein extracts were subjected to electrophoresis on 10% SDS-polyacrylamide gels and transferred to nitrocellulose membranes (Amersham Biosciences, Piscataway, NJ, USA). After blocking with 5% non-fat dry milk in TBS with 0.1% Tween-20 (TBS-T), the blots were incubated over-night with antibodies against Nrf2 (12721) and Lamin (2032) from Cell Signaling Technology; and β-actin (47778), followed by anti-rabbit (2004) or anti-mouse (2005) horseradish peroxidase-conjugated IgG (1:4,000) from Santa Cruz Biotechnology. The membranes were then submitted to 3 washes with 0.1% TBS-T after each incubation, and the proteins were detected using the ECL chemiluminescent detection system (Amersham Biosciences).

For *in vivo* experiments samples, C57BL/6 mice were injected with PBS, 1 pair of SGS or CoPP (1 mg/kg) in 10 uL PBS in both ears. Non-stimulated (naïve)mice were kept in a separate cage. After 24 h, both ears from each mouse were harvested, ground and homogenized in 1 ml PBS supplemented with protease inhibitors. Whole-tissue extracts were prepared in 1% Nonidet P-40 lysis buffer (1% Nonidet P-40, 150 mM NaCl, 20 mM Hepes (pH 7.5) supplemented with protease and phosphatase inhibitor cocktails (Sigma). Proteins were separated by SDS-PAGE, transferred to a nitrocellulose membrane and probed with Abs against HO-1 (ADI-OSA-110-J, Enzo, Ann Arbor, MI) and β-actin (47778) from Santa Cruz Biotechnology. Bands were visualized using an Image Quant 4010 apparatus (GE healthcare).

After exposure to sand fly bites, mice ears were harvested and dermal sheets separated with forceps and placed in RIPA buffer (Thermo Fisher Scientific, Waltham, MA, USA) supplemented with Halt™ Protease Inhibitor Cocktail (Thermo Fisher Scientific, Waltham, MA, USA) and PMSF (Sigma-Aldrich, St. Louis, MO, USA). The tissue was then submitted to mechanical disaggregation in a BD^TM^ Medimachine (BD Biosciences, Franklin Lakes, NJ, USA). Any remaining large particles were removed using a 50 μm Filcon filter (BD Biosciences, Franklin Lakes, NJ, USA). Whole tissue extract was kept on ice to insure complete lysis. After 15 min, the extract was centrifugated at 8,000 g for 10 min and the soluble fraction was collected. Proteins were separated by NuPAGE™ 4–12% Bis-Tris Protein Gels (Thermo Fisher Scientific, Waltham, MA, USA), transferred to a nitrocellulose membrane, and probed with antibodies against HO-1 (1:500, ab13248, abcam, Cambridge, MA, USA) and Hsp90 (1:500, #7964, Cell Signaling, Danvers, MA, USA). Bands were visualized by chemiluminescence detection using Azure c600 (Azure Biosystems, Dublin, CA, USA).

### Transient transfections and luciferase assays

To investigate the promoter activity, RAW-264.7 cells were plated in 24-well polystyrene plates (1 × 10^5^ cells per well) and transfected with 1μg of reporter plasmids using LIPOFECTAMINE 2000 reagent (Invitrogen, Carlsbad, CA, USA). The following plasmids were employed in the assays: 3xARE and Nrf2-WT. Luciferase activity was normalized using 40 ng of pRL-CMV plasmid (Promega Corp., Madison, WI, USA), 24 h after transfection the cells were treated. After treatment, cells rested for 24 h and were washed with PBS and lysed according to the Dual Luciferase System protocol (Promega Corp.), and analyzed using the GloMax^®;^-Multi detection system (Promega Corp., Madison, WI, USA). Positive controls consisting of cells stimulated with 10 mM DL-sulforaphane (SFN) (Sigma-Aldrich) were used to induce the activation of ARE and Nrf-2 gene expression.

### Cell sorting

After exposure to sand fly bites, mice ears were harvested and disinfected with ethanol. The dermal sheets were then separated using forceps and digested in PBS containing Liberase TL (Roche) at 37°C for 1 h. The digested tissue was further homogenized by mechanical disaggregation in a BD^TM^ Medimachine (BD Biosciences, Franklin Lakes, NJ, USA) and filtered in a 50 μm Filcon filter (BD Biosciences, Franklin Lakes, NJ, USA). Single cell suspensions were treated with anti-Fc (CD16/32) antibodies to block non-specific binding. After 15 min, cells were stained for Ly6C (clone AL-21; FITC; Biolegend), Ly6G (clone 1A8; APC-Cy7; Biolegend), CD11b (clone M1/70; PE-Cy7; Biolegend), F4/80 (clone BM8; PerCP; Biolegend) at a concentration of 1:100, and with LIVE/DEAD™ Fixable Yellow Dead Cell Stain Kit (1:1,000, Thermo Fisher Scientific, Waltham, MA, USA). Stained samples were sorted on a FACSAria-FUSION cell sorter using DIVA 6.1.8 software (BD Biosciences, San Jose, CA). Cell subsets were sorted at 4°C in chilled collection tubes supplemented with PMSF (Sigma-Aldrich, St. Louis, MO, USA). Collected cell subsets were pelleted down and lysed in RIPA buffer (Thermo Fisher Scientific, Waltham, MA, USA) for 15 min on ice. Immunoblotting against HO-1 (ab13248, abcam, Cambridge, MA, USA) and Histones H3 (#4499, Cell Signaling, Danvers, MA, USA) was carried out as previously described.

## Author contributions

NL, TD-V, WD, AV, LP, DC, CT, VSB, CdO, UL, NA, BA, CB, JV, SK, and VMB conceived and designed the study. NL, TD-V, WD, AV, LP, RF, PS-M, DC, CT, and CM performed the experiments. NL, TD-V, AV, VSB, CdO, UL, BA, CB, SK, and VMB contributed with data analysis. CM, VSB, UL, NA, BA, JV, SK, and VMB provided materials and infrastructural support. NL, TD-V, BA, SK, and VMB wrote and revised the manuscript.

### Conflict of interest statement

The authors declare that the research was conducted in the absence of any commercial or financial relationships that could be construed as a potential conflict of interest.

## Supplementary material

Supplemmentary Figure 1 Experimental exposure to *Lutzomyia longiplapis* sand flies induces HO-1 and Nrf2 protein expression at bite sites in healthy human volunteers. Immunohistochemistry of paraffin-embedded sections of skin specimens obtained by biopsy of the bite sites 48 h after sand fly exposure of a human volunteer. **(A)** HO-1 staining in sand fly-exposed skin, 400X. **(B)** Nrf2 staining in sand fly-exposed skin, 400X. Bar, 20 μm. Digital images were captured using a Nikon E600 microscope and an Olympus Q-Color 1 digital camera with the Image Pro Plus software.

Supplemmentary Figure 2 Feeding behavior of *Lutzomyia longipalpis* sand flies on mice ears. The number of sand flies that completed a blood meal after exposure to mouse ears. **(A,B)** Ears were used to assess HO-1 production. **(A)**, related to Figure 3A. **(B)**, related to Figure 3B. Bars represent the mean ± SEM. No statistical significant was observed among the different conditions by one-way ANOVA followed by Dunn's multiple comparisons test. Cumulative data is shown from two or three independent experiments.

Supplemmentary Figure 3 Gating strategy for sorting mice ear cells by flow cytometry. Ear cells recovered 24 h after exposure to 20 *L. longipalpis* sand fly bites were pooled and stained with antibodies for popuation sorting by flow cytometry. Live cells were gated on CD11b− cells (non-myeloid cells), CD11b+Ly6C+Ly6G+ (neutrophils), CD11b+Ly6C+Ly6G− (inflammatory monocytes) and CD11b+Ly6C−Ly6G−F4/80+ (resident macrophages). Data shown is representative of two independent experiments.

Supplemmentary Figure 4 Isotype control for IHC staining. **(A)** Isotype control for HO-1 staining, 400X **(B**) Isotype control for Nrf2 staining, 400X. Bar, 20 μm. Digital images were captured using a Nikon E600 microscope and an Olympus Q-Color 1 digital camera with the Image Pro Plus software.

## Abbreviations

SGS: salivary gland sonicate.
