# Supplementary figures and images for: Lutzomyia longipalpis Saliva Induces Heme Oxygenase-1 Expression at Bite Sites
Source: Front Immunol. 2018 Nov 28;9:2779. doi: 10.3389/fimmu.2018.02779 (PMC6279893; doi:10.3389/fimmu.2018.02779)

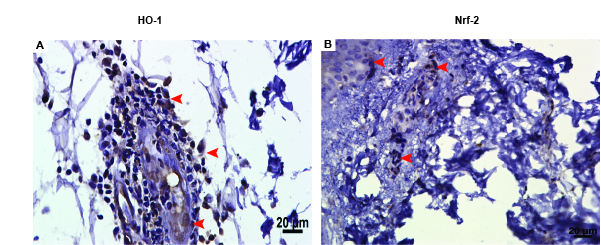

Supplement: Supplemmentary Figure 1 — Experimental exposure to Lutzomyia longiplapis sand flies induces HO-1 and Nrf2 protein expression at bite sites in healthy human volunteers. Immunohistochemistry of paraffin-embedded sections of skin specimens obtained by biopsy of the bite sites 48 h after sand fly exposure of a human volunteer. (A) HO-1 staining in sand fly-exposed skin, 400X. (B) Nrf2 staining in sand fly-exposed skin, 400X. Bar, 20 μm. Digital images were captured using a Nikon E600 microscope and an Olympus Q-Color 1 digital camera with the Image Pro Plus software. [file Image_1.TIF]

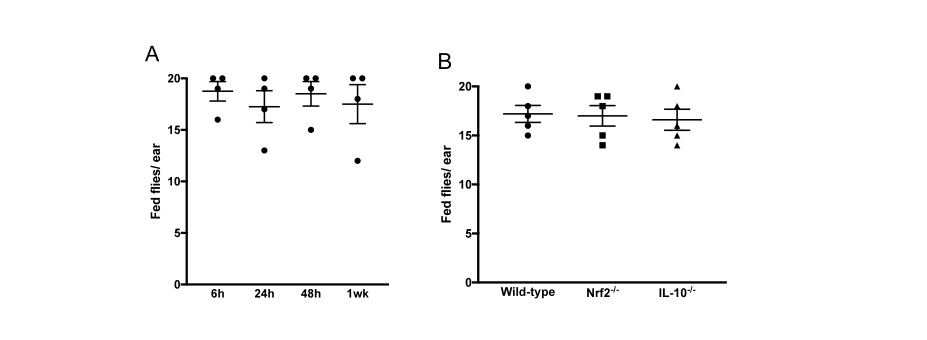

Supplement: Supplemmentary Figure 2 — Feeding behavior of Lutzomyia longipalpis sand flies on mice ears. The number of sand flies that completed a blood meal after exposure to mouse ears. (A,B) Ears were used to assess HO-1 production. (A), related to Figure 3A. (B), related to Figure 3B. Bars represent the mean ± SEM. No statistical significant was observed among the different conditions by one-way ANOVA followed by Dunn's multiple comparisons test. Cumulative data is shown from two or three independent experiments. [file Image_2.TIF]

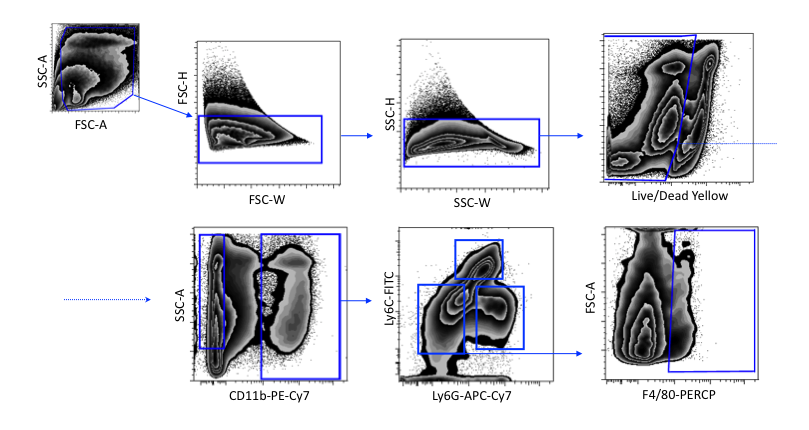

Supplement: Supplemmentary Figure 3 — Gating strategy for sorting mice ear cells by flow cytometry. Ear cells recovered 24 h after exposure to 20 L. longipalpis sand fly bites were pooled and stained with antibodies for popuation sorting by flow cytometry. Live cells were gated on CD11b− cells (non-myeloid cells), CD11b+Ly6C+Ly6G+ (neutrophils), CD11b+Ly6C+Ly6G− (inflammatory monocytes) and CD11b+Ly6C−Ly6G−F4/80+ (resident macrophages). Data shown is representative of two independent experiments. [file Image_3.TIF]

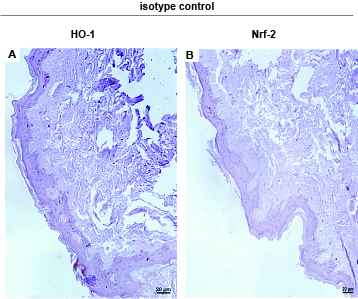

Supplement: Supplemmentary Figure 4 — Isotype control for IHC staining. (A) Isotype control for HO-1 staining, 400X (B) Isotype control for Nrf2 staining, 400X. Bar, 20 μm. Digital images were captured using a Nikon E600 microscope and an Olympus Q-Color 1 digital camera with the Image Pro Plus software. [file Image_4.TIF]
